# Enhanced Anticancer Response of Curcumin- and Piperine-Loaded Lignin-g-p (NIPAM-co-DMAEMA) Gold Nanogels against U-251 MG Glioblastoma Multiforme

**DOI:** 10.3390/biomedicines9111516

**Published:** 2021-10-21

**Authors:** Bilal Javed, Xinyi Zhao, Daxiang Cui, James Curtin, Furong Tian

**Affiliations:** 1 School of Food Science and Environmental Health, College of Sciences and Health, Technological University Dublin, Dublin, Ireland; D20127084@mytudublin.ie (X.Z.); james.curtin@tudublin.ie (J.C.); 2 Nanolab, FOCAS Research Institute, Technological University Dublin, Dublin, Ireland; 3 Department of Instrument Science and Engineering, National Center for Translational Medicine, Shanghai Jiao Tong University, Shanghai 200240, China; dxcui@sjtu.edu.cn

**Keywords:** caspase-3 apoptosis, cancer, drug delivery, glioblastoma multiforme, gold nanoparticles, immunostaining, kinetic drug release

## Abstract

Glioblastoma multiforme (GBM) is the most aggressive and commonly diagnosed brain cancer and is highly resistant to routine chemotherapeutic drugs. The present study involves the synthesis of Lignin-g-p (NIPAM-co-DMAEMA) gold nanogel, loaded with curcumin and piperine, to treat GBM. The ongoing study has the application potential to (1) overcome the limitations of drugs biodistribution, (2) enhance the toxicity of anticancer drugs against GBM, and (3) identify the drugs uptake pathway. Atom transfer radical polymerization was used to synthesize the Lignin-g-PNIPAM network, crosslinked with the gold nanoparticles (GNPs) to self-assemble into nanogels. The size distribution and morphological analysis confirmed that the drug-loaded gold nanogels are spherical and exist in the size of 180 nm. The single and combinatorial toxicity effects of curcumin- and piperine-loaded Lignin-g-p (NIPAM-co-DMAEMA) gold nanogels were studied against U-251 MG GBM cells. A cytotoxicity analysis displayed anticancer properties. IC_50_ of curcumin- and piperine-loaded gold nanogels were recorded at 30 μM and 35 μM, respectively. Immunostaining and Western blot analysis confirmed the protein expression of caspase-3 and cleaved caspase-3 in cells treated with drug-loaded nanogels. Kinetic drug release revealed 86% release of hybrid curcumin–piperine from gold nanogel after 250 min at pH 4. Atomic absorption spectroscopic analysis confirmed that the drug-loaded nanogels have better internalization or association with the cancer cells than the GNPs or nano-gels alone. Morphological studies further confirmed that the curcumin and piperine nanogels penetrate the cells via endocytic pathways and induce caspase-3-related apoptosis. The experimental evidence shows the enhanced properties of combinatorial curcumin–piperine gold nanogels (IC50: 21 μM) to overcome the limitations of conventional chemotherapeutic treatments of glioma cells.

## 1. Introduction

Glioblastoma multiforme is a fast-growing grade 4 brain glioma that usually develops in star-shaped glial or non-neuron cells (astrocytes and oligodendrocytes) that do not produce electrical impulses. Glial cells play a significant supportive role to neuron cells in the brain’s physiological functions [1]. GBM accounts for 60% of brain tumors and the median reported survival of GBM patients after its first diagnosis is unfortunately only about 16 months due to the unstoppable proliferation of glioma cells, poor diagnosis and disease prognosis, and a high-grade metastasis. Conventional cancer treatment of GBM includes the surgical removal of most of the cancerous cells or tumors followed by the chemotherapeutic administration of temozolomide combined with the radioactive treatment, which lasts from few weeks to several months. Unfortunately, there is no permanent cure or preventive medicine to kill all glioma cells to prevent their further proliferation, and tumor recurrence [2,3].

Curcumin (C_21_H_20_O_6_) is a polyphenol that is naturally available in turmeric (*Curcuma longa* L.), while piperine (C_17_H_19_NO_3_) is a biologically active and naturally available alkaloid that is abundant in pepper [4,5]. Previous studies [6,7] documented the significant anticancer potential of curcumin and piperine by the induction of apoptosis in cancer cells and their preventive role in angiogenesis. The anticancer potential of curcumin and piperine is attributed to the ability to target various signaling molecules at molecular and cellular levels [6]. Curcumin and piperine perform their cytotoxic activities by triggering cells’ defense response through the generation of reactive oxygen species (ROS). Active ROS generation results in triggering caspases-induced apoptosis, enhanced mitochondrial membrane permeability, disruption of electron transport chain, inhibition of enzymes’ activity, nucleic acid fragmentation, hampering of the transcription, DNA replication pathways, and the disassembly of the plasma cell membrane [7]. These are a few among the major pathways by which curcumin and piperine achieve apoptosis of the cancerous cells [8,9].

However, the major limitations to using curcumin and piperine as an active therapeutic to treat brain glioma are the poor aqueous solubility and the reduced bioavailability which limit their efficacies [6,10]. Recent advances in the field of nanotechnology provided an opportunity to design smart drug delivery systems having nano-sizes (1 and 100 nanometers), uniform shapes, enhanced biodistribution, and adsorption [11,12,13,14].

Nanogels are an advanced type of nanocarriers that are a nanosized network of crosslinked or entangled polymers, stabilized by physical and chemical forces. The nanogels have abilities to self-assemble with hydrophilic or hydrophobic drugs and act as a novel platform to carry therapeutics and to deliver them to their targeted sites [15,16]. The nanogels have specific physical, chemical, and biological features that include the specific surface tension, surface area, enhanced absorption, controlled release, inner space or cavity, bioavailability, or circulation and stimuli-responsive activity which makes them suitable candidates for the delivery of drugs to the GBM cells. The use of nanogels in combinatorial nanotechnological approaches has synergistic effects which result in enhancing the cytotoxic potential [17,18].

Designing nanosized formulation to carry curcumin and piperine has the potential to address their limitations and can also have efficient therapeutic potential with improved drug efficacy and bioavailability. A great effort has been done to examine the synergistic effects of a monoclonal antibody (CD68) combined with curcumin and it was confirmed experimentally that the synergistic anticancer and cell death activity increased 120-fold in lines T98G and U87MG for GBM cells [19]. Another study confirmed the synergistic anticancer role of curcumin and piperine to suppress the proliferation of diethylnitrosamine (DENA)-induced hepatocellular carcinoma (HCC) in rats [20]. Additionally, the gold nanoparticle has been a promising contrast agent for in vivo imaging due to its high density and plasmonic property [19]. However, there is no report on the combinatorial application treatment of curcumin and piperine with the gold nanoparticles’ crosslinked nanogels against the GBM cells. 

**Scheme 1:**
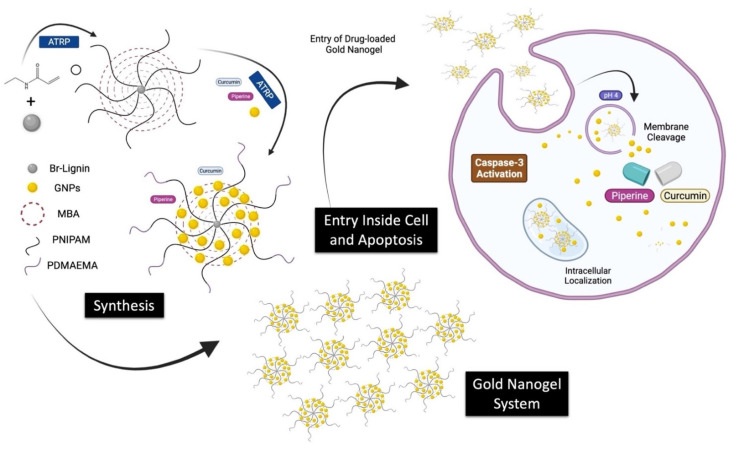
Layout of the study.

The present study is designed to use atom transfer radical polymerization (ATRP) to synthesize the Lignin-g-PNIPAM crosslinked gold nanoparticles (GNPs) network to self-assemble into nanogels. The nanogel of Lignin-g-PNIPAM was immersed in GNPs solution. The PNIPAM blocks with bromides on their ends were acting as sites of growing up the PDMAEMA blockchains and were used as an initiator of the second reaction of ATRP polymerization. The final obtained drug delivery system was equipped with the targeting agents while having the dual ability to load curcumin and piperine. This study was envisaged to elaborate the single or combinational toxicity effects of curcumin and piperine with gold Lignin-g-p (NIPAM-co-DMAEMA) nanogel against U-251 MG GBM cells. The detailed layout of the study is given in Scheme 1. 

## 2. Materials and Methods

### 2.1. Synthesis and Self-Assembly of Lignin-g-P (NIPAM-co-DMAEMA) Gold Nanogel Drug Delivery System

The atom transfer radical polymerization (ATRP) was performed in two separate steps for the fabrication of Lignin-g-PNIPAM followed by the synthesis of GNPs-b-PDMAEMA. The synthesis of polymers was monitored by performing the ^1^H NMR (Nuclear Magnetic Resonance) (Figures S1–S4). Brominated lignin was used as an initiator in the reaction of ATRP polymerization. During this study, the constant ratio of H_2_O and N, N-Dimethylformamide (3.5:1.5) as solvent and monomers of N-Isopropylacrylamide (NIPAM) and N-N’ methylene bisacrylamide (MBA, cross-linker agent)/CuBr/ligand/macroinitiator concentration have been used to obtain nanogel of Lignin-g-PNIPAM with the desirable properties [21].

Frozen micelle synthesis was achieved by stirring the copolymer powder in an HNO_3_ aqueous solution at pH 1 and 95 °C for 1 day to obtain a transparent polymer solution, which was then plunged into an ice bath. The chains were then self-assembled into frozen micelles with a polystyrene core surrounded by a corona of PDMAEMA chains extending in the solvent [21].

Gold nanoparticles were synthesized by the reduction of chloroauric acid with the help of trisodium citrate [21,22]. The colloidal gold was prepared as HAuCl_4_ (100 mL, 0.01% [*w/v*]) (Sigma-Aldrich, USA) was heated to boiling. This was followed by the rapid addition of 5 mL of trisodium citrate (1% [*w/v*]) (Sigma-Aldrich, St. Louis, MO, USA) while the mixture was stirred at high speed and heated for 30 min. After natural cooling at room temperature, colloidal gold was filtered through a 0.22 µm membrane and stored in the dark at 4 °C for future use. Colloidal gold (1 mL) solution was adjusted to pH 1 and mixed with a 100 mL of polymer concentration of 0.5% *w/w* at 95 °C [21].

**Figure 1:**
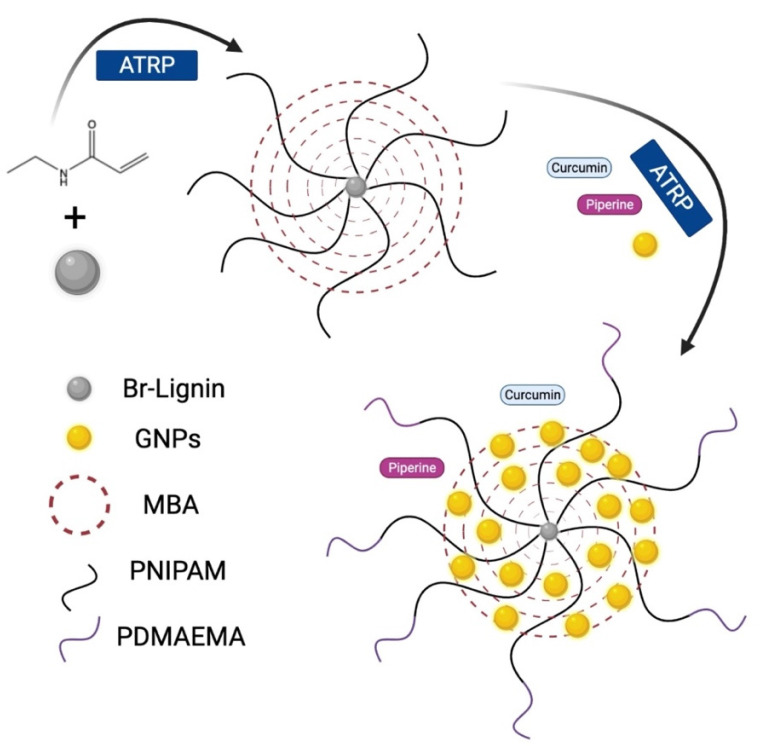
Schematic representation of the synthesis of Lignin-g- p (NIPAM-co-DMAEMA) gold nanogels.

The anticancer drugs curcumin or piperine were dissolved at 20 mM in 20 mL of acetone solution. The solution was mixed with 100 mL of gold nanogel. Then acetone was evaporated to prepare the curcumin-loaded gold nanogel mixture, piperine-loaded gold nanogel mixture, and hybrid curcumin–piperine-loaded gold nanogel mixtures. The schematic representation of the synthesis of gold nanogel is given in Figure 1.

### 2.2. Physical and Optical Characterization of Drug Carrying Lignin-g-P (NIPAM-co-DMAEMA) Gold Nanogels 

The characterization of the theranostic nanogels and GNPs was performed by using various analytical instruments. The nanogels of various types were suspended separately in Dulbecco’s Modified Eagle’s Medium high glucose (DMEM) cell culture medium without any additional supplementation and were analyzed for their morphology, size, and surface charge.

The morphology of drug-loaded nanogels and GNPs was assessed by using a scanning electron microscopic (SEM) (Hitachi SU6600, Tokyo, Japan) instrument. The sample was prepared by pipetting a total of 10 μL of the colloidal solution in methanol onto prewashed silicon substrates and spin coated at a speed of 1000 rpm for 20 s. The samples were dried in air and characterized by electron microscopy using a Hitachi SU6600 FESEM instrument at an acceleration voltage of 25 kV. Images were captured by using the SEM detector [23].

### 2.3. Particle Size Analysis of Drug Carrying Lignin-g-P (NIPAM-co-DMAEMA) Gold Nanogels

The dimensions of each gold nanogels were assessed by using the dynamic light scattering (DLS) instrument and was performed via a NanoZetasizer ZS analyzer (Malvern Instruments, Worcestershire, UK). The colloidal sample solution for the size measurements was prepared by dispersing 100 μg/mL of gold nanogels in the DMEM-high glucose (Dulbecco’s Modified Eagle’s Medium-high glucose, Merck, Arklow, Ireland) cell culture medium in DTS0012 cuvettes. The refractive index (RI) of 1.6 was used for gold nanogels. The viscosity of the sample colloidal solutions was measured at 25 °C with the help of a viscometer SV-10 (A&D Instruments Ltd., Abingdon, UK) before the DLS analysis was performed, and the recorded values were used for the hydrodynamic size estimation of gold nanogels. The samples were equilibrated at 25 °C for three minutes before each measurement. Additionally, the surface charge in terms of zeta potential of each gold nanogel was also measured in supplemented DMEM cell culture medium by a NanoZetasizer ZS analyzer (Malvern Instruments, Worcestershire, UK). The DTS1060C clear disposable zeta cells were used as a sample holder for the measurement of zeta potential, and the measurements were performed at 5 V. The data was recorded in the form of three independent measurements [24].

### 2.4. Determination of Curcumin and Piperine Concentration in Nanogel

The curcumin- and piperine-loaded nanogel and the standards were measured at 430 nm and 340 nm wavelength using a Perkin Elmer Lambda 900 UV/VIS/NIR Spectrometer to determine curcumin and piperine concentrations, respectively. The standard curves were prepared from the values of standards. The curcumin and piperine concentrations of the curcumin- and piperine-loaded nanogel were estimated by the read-out of the absorbance intensity and corresponding concentration on the standard curve. The number of curcumin- and piperine-loaded nanogel and gold nanogel concentrations were alternatively defined as particles per mL with the help of the NanoSight NS300 instrument (Malvern Instruments, Worcestershire, UK) NTA system. Video capture and analysis were performed by using the NTA software (version 3.4, West Bengal, India).

### 2.5. Determination of Kinetic Drug Release from Drug Carrying Lignin-g-P (NIPAM-co-DMAEMA) Gold Nanogels

Kinetic drug release of curcumin and piperine from gold nanogels was determined in various combinations of nanogels with curcumin and piperine at pH 4 and pH 7.4. The enzymes show activity in acidic conditions, requiring the organelle to maintain an optimal luminal pH between 4 and 5. pH 4 stimulated the nanogel system under lysosome and stomach conditions. pH 7.4 stimulated the nanogel system under cell culture and intestinal conditions. The kinetic release of the drug was determined by performing HPLC analysis. Curcumin and piperine were analyzed by the HPLC column C_18_, with UV detection at 425 nm. The mobile phase was acetonitrile and water (50:50 *v/v*) acidified with 2% of acetic acid at a flow rate of 1.2 mL/min. The curve range was linear for the receptor solution at the concentration range of 0.5–75 μg/mL [25,26].

### 2.6. U-251 MG Glioblastoma Cell Culture Maintenance

The U-251 MG human brain glioblastoma astrocytoma cancer cells were cultured in DMEM-high glucose medium supplemented with 10% of FBS (Fetal Bovine Serum, Merck, Darmstadt, Germany) and maintained at 37 °C in an incubator with a humidified atmosphere of 5% (*v/v*) CO_2_ [27].

### 2.7. Treatment of U-251 MG Glioblastoma Cell with Drug Carrying Lignin-g-P (NIPAM-co-DMAEMA) Gold Nanogels 

The colloidal gold nanogels were diluted in a culture medium with a curcumin or piperine concentration of 0.6, 1.25, 3, 6, 12.5, 25, 50, 100, 250, 500, and 1000 μM. The same amounts of nanogel and gold nanogel were loaded for cell culture at the same time, such as 6 × 10^5^, 1.3 × 10^6^, 2.6 × 10^6^, 5.2 × 10^6^, 1.0 × 10^7^, 2.1 × 10^7^, 4.2 × 10^7^, 8.4 × 10^7^, 1.7 × 10^8^, 3.4 × 10^8^, 6.7 × 10^8^ particle/mL. The treated cells were observed under the microscope before the addition of alamarBlue^®^ in order to rule out the potential of incorrect plating of the cells [28].

The statistical analysis was performed by using the GraphPad Prism^®^ software (San Diego, CA, USA). Non-linear regression analysis was performed by the prism to plot a dose–response curve and to graphically observe the relationship between the drug and the percentage viability of the cells. It was also used to generate an IC_50_ of the drug, which in this instance is an inhibitor [29].

### 2.8. Apoptosis Immunostaining of U-251 MG Glioblastoma Cells Treated with Drug Carrying Lignin-g-P (NIPAM-co-DMAEMA) Gold Nanogels 

The U-251 MG cells were seeded at a density of 4 × 10^4^ cells/cm^2^ and treated with 0.2 mg/mL of gold nanogels for 72 h in 8-well chamber slides. The cells were fixed by using -20 °C methanol for 10 min and with -20 °C acetone for 1 min on microscope coverslips. The coverslips were washed twice with PBS solution and then blocked with PBS solution containing 0.1% of BSA (Bovine Serum Albumin) for 10 min at room temperature in the dark. The samples were incubated for 60 min with an anti-cleaved Caspase-3 antibody [E83-77] (Abcam) in PBS solution containing 1% of BSA and then washed three times in PBS solution. Following this, the sample was incubated for 30 min with Alexa 546 anti-rabbit (A11035, Invitrogen, Dublin, Ireland) as the secondary antibody and a green phalloidin probe (A12379, Invitrogen, Dublin, Ireland; to denote the cell F-actin cytoskeleton) at a 1:40 dilution in PBS solution containing 1% of BSA. The samples were subsequently washed three times in PBS solution. After staining the nuclear region with 4′,6-diamidino-2-phenylindole dye (DAPI), one drop of ProLong^®^ Gold Antifade mountant reagent (Invitrogen, Dublin, Ireland) was added onto the coverslips before they were carefully inverted onto glass microscope slides. The samples were then imaged by using laser scanning microscopy (Carl Zeiss 510). Four images per sample were captured to gain a representative understanding of the onset of apoptosis in U-251 MG cells following the gold nanogel carrier exposure. Subsequently, the image analysis was performed with ImageJ^®^ software to quantify the fluorescent density, according to the procedure described in a study carried out by reference [22].

For the Western blot analysis, anti-rabbit anti-caspase-3 and anti-cleaved caspase-3 antibodies were used that recognizes the caspase-3 and cleaved CASP3 fragment (Amersham-Pharmacia Biotech, Buckinghamshire, UK; E83-77, Abcam, 50,000:1 dilution). The Western blot was visualized by using a chemiluminescence kit (ECL, Amersham-Pharmacia Biotech) and Western blots were transferred to Bio-RAD Imaging, Hercules, CA, USA. Densitometric analysis was performed by using ImageJ^®^ software (version 1.45s) and protein expressions were normalized to the density of β-actin, respectively (100%). The analysis was performed in triplicates and from three independent experiments (*n* = 3).

### 2.9. Estimation of Internalized Drug Carrying Lignin-g-P (NIPAM-co-DMAEMA) Gold Nanogels by Using Atomic Absorption Spectroscopy 

The Atomic absorption spectroscopic (AAS) analysis was employed to quantify the total gold mass per dish using an atomic absorption spectrometer (SpectrAA200, Varian, Palo Alto, CA, USA) with direct comparison with a commercially purchased AAS gold standard (TraceCERT, Fluka, Arklow, Ireland). Cells were exposed to 1 mg/mL of gold nanogels in a 10 mL of medium (10 mg of NPs) via suspension method, in a Petri dish with 10 cm diameter. After 72 h of exposure at 37 °C and 5% of CO_2_ atmosphere, the cell culture medium was removed and attached cells were thoroughly washed with phosphate buffer saline (PBS) solution. The loosely associated gold nanogels to the cell membrane were further washed with the PBS solution and subsequently detached by trypsinization by using trypsin (a proteolytic enzyme that is responsible for breaking proteins and dissociates adherent cells from the vessel in which they are being cultured). Following the trypsinization, the cells were washed three times with the PBS solution via centrifugation at 1400 rpm for 10 min. The cells were then counted using a Z2 coulter counter (Beckman Coulter, Brea, CA, USA) and subsequently air-dried for 24 h at room temperature. Following the incubation period, the cell samples were dispersed in 8 mL of water and then sonicated for 30 min (135 W) at room temperature to ensure a uniform distribution of gold in the sample before AAS analysis. The total mass of associated gold per sample (either internalized or tightly bound to the cell surface) was determined by three independent measurements and presented as the average absorbance [22,27].

### 2.10. Estimation of Internalized Drug Carrying Lignin-g-P (NIPAM-co-DMAEMA) Gold Nanogels by Using Transmission Electron Microscopy

The individual volume of single gold nanogels was observed by using TEM analysis, allowing for the further estimation of the total number of gold nanogels per dish. The sub-cellular deposition of gold nanogels was determined by using the conventional TEM instrument. The U-251 MG cells were seeded at a density of 5 × 10^5^ cells in a 30 mm polystyrene dish for 24 h at 37 °C, and 5% of the CO_2_ atmosphere was maintained. Following the increased cell density, the concentration of gold nanogels was also increased to serve the same nanoparticles per cell concentration. This was performed due to the inherent nature of TEM sample processing, in which cell numbers can be significantly reduced via the dehydration, washing, and staining steps during sample preparation. The cells were subsequently incubated with 0.1 mg/mL (3 mL volume) of gold nanogel for 72 h. It was also considered important to use the same concentration of the sample as used for AAS analysis. Following the exposure period, the cells were washed three times with the PBS solution and then fixed overnight at pH 7.4 by using 2% of paraformaldehyde, 2.5% of glutaraldehyde, and 0.15 M of sodium phosphate. Ultrastructural analysis and photo-microscopy were performed with a JEOL JEM-2100 electron microscope instrument. At least five independent fields of view from three individual samples were captured in order to provide a representative qualitative understanding of the interaction between the different shaped gold nanogels and the U-251 MG cells [22,23].

### 2.11. Statistical Analysis and Data Management 

The experiments were performed in triplicate and the data were presented as the mean and standard deviation. MS Excel^®^ spreadsheets were used to arrange data. The significant differences between the two groups were evaluated by using Student’s *t*-test, or between multiple groups via two-way analysis of variance (ANOVA). The regression analysis was performed, and Tukey’s multiple comparison post hoc test was used to identify the source of variance. The statistical analyses were performed by using the GraphPad Prism^®^ 9.10 software (GraphPad Software, San Diego, CA, USA). The alpha value was set to 0.05 to indicate the significant differences.

## 3. Results and Discussions

### 3.1. Synthesis of Gold Nanoparticles and Drug Carrying Lignin-g-P (NIPAM-co-DMAEMA) Gold Nanogels

Nanogels play a promising role in crosslinking polymers and gold nanoparticles to encapsulate drugs, and self-assemble to carry therapeutics to their designated locations. Nanogels also play a significant role in increasing the biodistribution of the drugs by promoting dissolution in an aqueous medium [10,16,30]. The synthesis of Lignin-g-p (NIPAM-co-DMAEMA) nanogels crosslinked with gold nanoparticles provides a novel platform to overcome the limitations of the bioavailability of curcumin and piperine and enhance the therapeutic efficacy and the effectiveness of the drug against brain glioma cancer cells. The synthesis of nanogels was monitored by using ^1^H NMR analysis and is provided in Figures S1–S4.

The gold nanoparticles were synthesized by using the chemical reduction of the chloroauric acid into gold nanoparticles. The association of the GNPs with the lignin polymers resulted in the crosslinking to form the self-assembled gold nanogels. The two-step atom transfer radical polymerization resulted in the synthesis of Lignin-g-PNIPAM and GNPs-b-PDMAEMA, while the N-N’ methylene bisacrylamide (MBA) was used as a crosslinker chain. The detailed layout of the synthesis of the gold nanogel therapeutic system is explained in Figure 1.

The curcumin- and piperine-loaded gold nanogel synthesized in this study showed curcumin concentrations on average 0.37 mg/mL, while the maximum piperine content of the curcumin and piperine-loaded gold nanogel was 0.29 mg/mL. The number of curcumin- and piperine-loaded nanogel achieved 6.7 × 10^8^ particles. A total of 1 mM curcumin concentration of curcumin-loaded gold nanogels corresponds to approximately 6.7 × 10^8^ particles per mL, and 1 mM piperine concentration of piperine-loaded gold nanogels corresponds to 6.7 × 10^8^ particles per mL.

### 3.2. Physical and Optical Characterization of Drug Carrying Lignin-g-P (NIPAM-co-DMAEMA) Gold Nanogels

**Figure 2:**
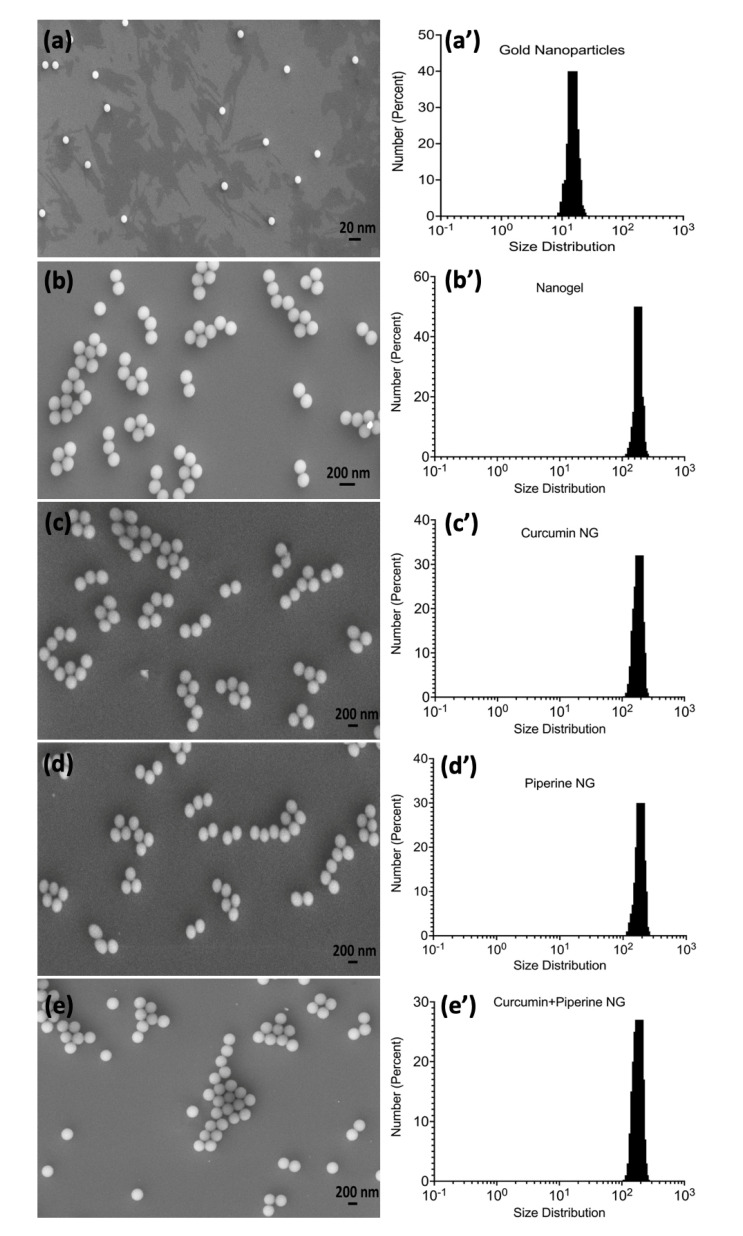
A scanning electron microscopic images and size distribution analysis of nano-formulations; (**a**) gold nanoparticles; (**b**) gold nanoparticles and nanogel; (**c**) curcumin-loaded gold nanogel; (**d**) piperine-loaded gold nanogel; (**e**) hybrid curcumin–piperine-loaded gold nanogel; (**a’**) size distribution analysis of gold nanoparticles; (**b’**) size distribution analysis of nanogel; (**c’**) size distribution analysis of curcumin-loaded nanogel; (**d’**) size distribution analysis of piperine-loaded nanogel; (**e’**) size distribution analysis of curcumin–piperine-loaded nanogel.

SEM image analysis was performed to determine the shape and size of the gold nanoparticles, gold nanogels, and their various therapeutic combinations with curcumin and piperine (Figure 2). It was observed that the gold nanoparticles are spherical or globular while they were uniformly dispersed and separated from each other (Figure 2a). The assemblage of gold nanoparticles with the nanogels was also represented in the form of small spheres. However, the addition of nanogels resulted in small clusters of nanoparticles being held together with the help of the nanogel polymeric structure (Figure 2b). The combinatorial drug-loaded gold nanogel with curcumin and piperine in singular and hybrid combinations was also spherical and existed in the form of small clusters (Figure 2c–e). SEM image analysis further showed that the GNPs exist at 15 nm in size, while most of the nanoparticles were uniformly dispersed. However, the association of the drugs with the nanogels resulted in the swelling of the gold nanogels, and their size increased to 200 nm while the nanoparticles were observed to be uniformly polydispersed in Figure S5. 

The particle size analysis in Brownian motion was performed to measure the hydrodynamic diameter of GNPs, gold nanogels, and piperine-loaded nanogel, curcumin-loaded nanogel, and curcumin–piperine-loaded nanogel (Figure 2 and Table 1). The GNPs have an average diameter of 15 nm (Figure 2a’). The average diameter of the nanogel was recorded at 160 nm, while it was examined that most of the nanogels in the system have a size of 180 nm (Figure 2b’). The curcumin-loaded nanogel, piperine-loaded nanogel, and curcumin–piperine-loaded nanogel had the average hydrodynamic diameter, recorded at 201 nm, 198 nm, and 206 nm, respectively (Figure 2c’–e’). The hydrodynamic diameter of GNPs and nanogels was recorded higher than the average size recorded by the SEM image analysis. The higher hydrodynamic diameter collectively represents the size of the gold nanoparticle’s metallic core and the associated biochemical functional groups which hang out from the surface of the metallic core.

The GNPs, gold nanogel, and curcumin- and piperine-loaded gold nanogels were found to exhibit a zeta (ζ) potential in the range of 23.21–29.67 mV, recorded in cell culture medium. The magnitude of zeta potential plays a promising role to determine the electrostatic interactions between the therapeutic nanogels [31]. The zeta potential values were strongly positive, which suggests that the presence of polymeric chains of self-assembled Lignin-g-P (NIPAM-co-DMAEMA) gold nanogels form complexes with the positively charged corona of PDMAEMA chains extending in the solvent. The zeta potential values less than 30 mV represent that the nanogels have sufficient electrostatic repulsive force to maintain better physical colloidal stability among nanogels [32].

### 3.3. Determination of Kinetic Release of Curcumin and Piperine from Lignin-g-P (NIPAM-co-DMAEMA) Gold Nanogels

Drug release kinetics is considered an important step in a drug discovery process. The drug release is a mechanistic process that involves the initial migration of drug solute components in the polymeric system to the outer side of the polymer which is then followed by the release of the drug in the releasing medium [7]. There are many biochemical and physiological mechanisms that not only control but also influence the process of drug release such as the polymeric nature of the drug, the type of the solute and releasing medium. Drug release kinetics play a promising role to determine the mass transport mechanisms which influence the release of the drug [15].

**Figure 3:**
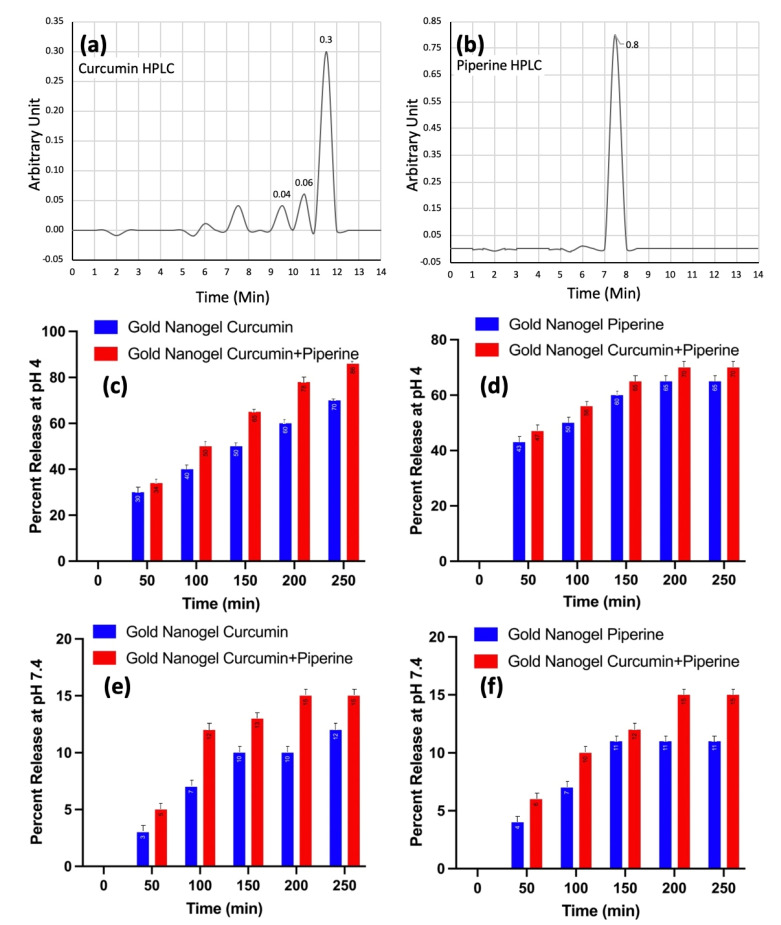
Determination of drug release kinetics; (**a**) curcumin HPLC; (**b**) piperine HPLC; (**c**) kinetic release of curcumin from nanogels at pH 4; (**d**) kinetic release of piperine from nanogels at pH 4; (**e**) kinetic release of curcumin from nanogels at pH 7.4; (**f**) kinetic release of piperine from nanogels at pH 7.4.

The present study involves a detailed explanation of the kinetic release of the drug such as curcumin and piperine from the drug-loaded gold nanogels. The HPLC chromatograms manifested the amount of curcumin (Figure 3a) and piperine (Figure 3b) released from the gold nanogels. The kinetic release of the drugs such as curcumin and piperine, separately and in combined co-treatment was studied at pH 4 and pH 7.4. It was observed that the drug release efficiency was very high at acidic pH 4 and the drug release response was directly proportional to the time. The drug release percentage increased gradually over time. The drug release kinetics was recorded at 70% and 86% after the passage of 250 min when curcumin-loaded gold nanogel and curcumin–piperine-loaded gold nanogels were used, respectively (Figure 3c). The piperine-loaded gold nanogel exhibited 65% of drug release kinetics after 250 min of treatment (Figure 3d).

The drug release efficiency was decreased gradually at pH 7.4. It was observed that the curcumin-loaded gold nanogel and piperine-loaded gold nanogel exhibited 12% and 11% of drug release at 250 min (Figure 3e,f).

Figure 3 explains that the drug release kinetics is higher at the acidic pH compared with the neutral pH. The specific nature of the nanogels to release drugs in response to a pH shows their pH-responsive abilities to trigger the release of drugs. A previous study showed that the higher release of drugs at the acidic pH is due to the swelling of the nanogel. The release of the drug from the nanogel depends on a combination of diffusion factors, the process of degradation of the drug, the nature of the surrounding solvent and the pH [7].

The PDMAEMA chains in the lignin-based nanogels collapsed to smaller dimensions at a lower pH [33]. The release of the curcumin–piperine from the gold nanogel was higher at different time intervals and pH, compared with the curcumin-loaded gold nanogel and piperine-loaded nanogel separately. The kinetic release of drugs at the acidic pH plays a significant role in cancer medicines due to the acidic nature of the cancer cells [2,3]. The high kinetic release of the drugs from the drug-loaded nanogel at a low pH may help to control the release of the drug in cancer cells following endocytosis and trafficking to the lysosomal compartment.

### 3.4. Cytotoxicity Analysis of Drug Carrying Lignin-g-P (NIPAM-co-DMAEMA) Gold Nanogels and Curcumin and Piperine Co-Treatment Approach against U-251 MG Glioblastoma Cells

Glioblastoma multiforme is a high-grade brain cancer of glial cells and is a leading cause of death due to its nature to resist chemotherapeutic and radioactive treatment. However, the major treatment challenges include the difficulties that the chemotherapeutic medicines face to cross the blood-brain barrier, which results in disease recurrence and a negative treatment success rate [34].

**Figure 4:**
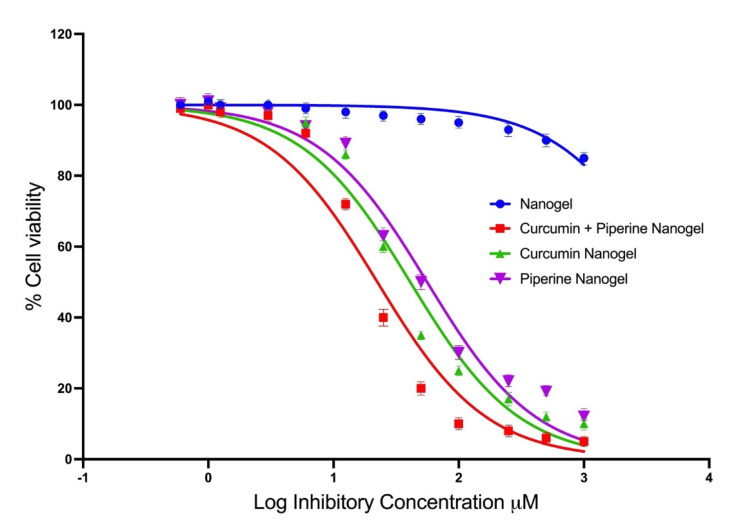
The percentage cell viability of the U-251 MG glioblastoma cells treated with piperine and curcumin-loaded nanogels.

Figure 4 represents the percentage cell viability of the U-251 MG glioblastoma cells treated with piperine and curcumin. The cell viabilities higher than 100% are presumed to have been acquired as a result of overexposure of alamarBlue® and excitation of response. At a 100 μM concentration of piperine nanogel, cell viability of 37.50% was observed, and at a concentration of 50 μM, the cell viability of 67.90% was recorded. This proves that piperine itself exhibits promising anticancer properties against the U-251 MG glioblastoma cell line.

It was observed from the IC_50_ values in Figure 4 that the cytotoxicity results of the curcumin–piperine hybrid nanogel gave the highest average cytotoxicity of all the drug-loaded nanogels with an average IC_50_ value of 21 μM. The curcumin gold nanogel followed the second-highest cytotoxicity, with an average IC_50_ value of 30.72 μM. The piperine nanogel gave the lowest cytotoxicity, with an IC_50_ value of 35.04 μM. The combination index (CI) of hybrid curcumin–piperine gold nanogel was reported at 0.886. A CI value less than 1 shows the synergistic response of the anticancer drug. The recorded CI value at 0.886 shows the mild synergistic response and enhanced anticancer activity. The isobologram is provided in Figure S6. Overall, curcumin exposure displayed a significant level of cytotoxicity to U-251 MG cells (*p* < 0.0001). The *p* values obtained are <0.0001. This concludes that there is a high statistical significance to the greatest degree between the different curcumin and piperine drugs and their concentration.

The findings of the present study show that the nanogels act as a vehicle to carry therapeutics to the cells to enhance their biodistribution and cytotoxicity against glioblastoma cells. A study was performed earlier by Thani et al., who achieved the cell death of U-251 MG glioma cells from curcumin at concentrations of 25 μM [35]. The IC_50_ value of the piperine nanogel was recorded at 35 μM. A previous scientific study recorded an IC_50_ value at 24 μM by using piperine as an anticancer drug to treat human brain cancer cell lines (Hs 683) [36]. However, the enhanced effects of piperine and curcumin were potentially higher and the IC_50_ value of curcumin and piperine hybrid gold nanogel was recorded at 21 µM.

The application of nanogels not only contribute to promoting bioavailability but also function to enhance their toxicity. The effects of a co-treatment of piperine and curcumin nanogels on the cell viability of U-251 MG glioblastoma cells were remarkable. The assembly of curcumin and piperine into nanogel provides advantages to enhance the bioavailability and biodistribution of cancer drugs to the targeted cancer glioma cells. The presence of piperine has the advantage to increase the cytotoxic effects of curcumin through a co-treatment approach [37,38]. The hybrid drug-loaded nanogels activate the effector caspases such as caspase-3 apoptotic pathways either directly or via the mitochondria-mediated apoptosome. The expression of caspase-3 in U-251 MG cells was further investigated by immunoblotting assay.

### 3.5. Apoptosis Immunostaining and Caspase-3 Expression of U-251 MG Cells 

**Figure 5:**
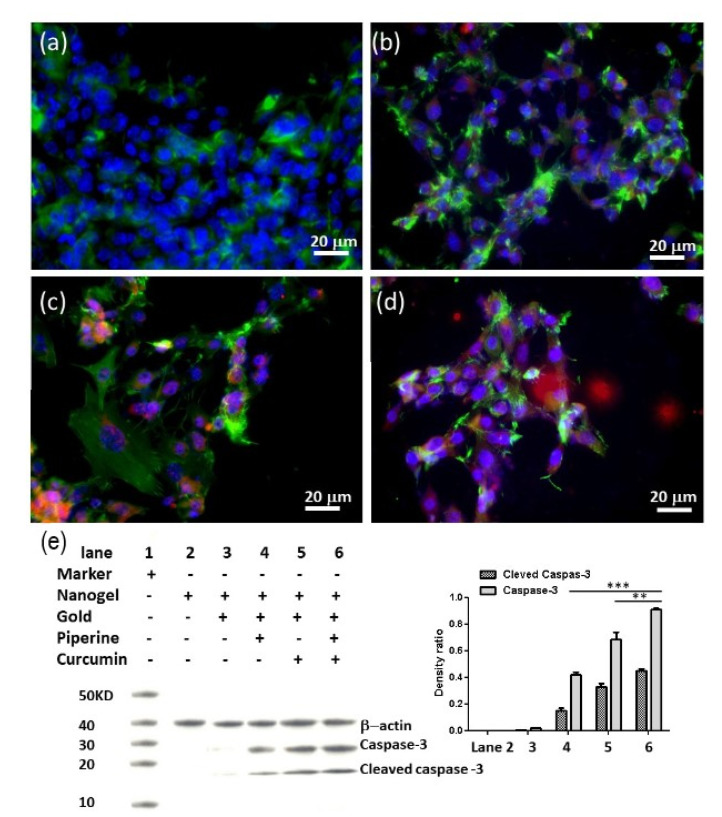
Immunoblotting of U-251 MG cells under nanogel treatment. Laser scanning microscopy images show U-251 MG cells treated with (**a**) nanogel, (**b**) piperine-loaded nanogel, (**c**) curcumin-loaded nanogel, (**d**) curcumin–piperine-loaded nanogel after 72 h at 21 µM concentration. (**e**) Western blot and histogram of density ratio of caspase-3/β-Actin and density ratio of cleaved caspase-3/β-Actin. (** *p* < 0.01, *** *p* < 0.001). Color code: Caspase-3: Red; F-actin: Green; Cell nuclei: Blue.

Immunoblotting of U-251 MG cells was performed after the treatment with drug-loaded gold nanogel. The laser scanning microscopic image analysis of U-251 MG cells was performed after 72 h of incubation of glioma cells at 21 µM of curcumin and piperine gold nanogels. It was observed that the treatment of U-251 MG cells with the nanogels resulted in the alteration of cellular cytoskeletal protein F-actin which induced the caspase-3 induction of apoptotic pathways (Figure 5a–d). The Western blot analysis results revealed the active protein expression of caspase-3, cleaved caspase-3 and β-actin. The nanogel was used as a negative control. The use of a nanogel alone or gold nanogel as an anticancer drug against the GBM cells did not show the activity of caspase-3. However, the piperine-loaded gold nanogel showed the expression of caspase-3 protein with a very thin band while a dense band was observed when curcumin-loaded nanogel was used as an anticancer drug against GBM cells. The higher expression of caspase-3 was observed when the hybrid curcumin–piperine-loaded nanogel was used (Figure 5e). There are statistical differences between hybrid curcumin–piperine-loaded gold nanogel with curcumin or piperine-loaded gold nanogel (** *p* < 0.01, *** *p* < 0.001).

### 3.6. Estimation of Gold Association with U-251 MG Glioblastoma Cells in Various Nano-Formulations by Using Atomic Absorption Spectroscopy

Atomic absorption spectroscopy plays a promising role to quantify the gold mass in nanoparticles and nanogels associated with cancer cells. However, the AAS analysis has one limitation as it cannot distinguish between the nanomaterials associated with the cells or internalized the cells [22]. The detailed AAS analysis results are represented in Table 2. The total Au mass per dish was decreasing after their assemblage with nanogels and drugs. It was observed experimentally that the GNPs and gold nanogels are associated with the glioblastoma cells at the same rate of 8 × 10^5^ per cell. There was a similar uptake of gold nanoparticles’ mass per cell. However, the loading of curcumin and piperine on nanogels resulted in a marked increase in the amount of gold per dish, and values were recorded at 1.15 × 10^6^ and 1.30 × 10^6^ mass per cell, respectively. The nanogel as a drug carrier has shown the enhanced effects of curcumin–piperine nanogels to co-treat the resilient glioma brain cancer more accurately and precisely and does not accelerate gold nanoparticle up-taking. The gold nanoparticles of curcumin–piperine nanogels localization or internalized in cells were further investigated by using TEM imaging analysis.

### 3.7. Visualization of Internalization of Curcumin- and Piperine-Loaded Gold Nanogels in U-251 MG Glioblastoma Cells by Using TEM Analysis

**Figure 6:**
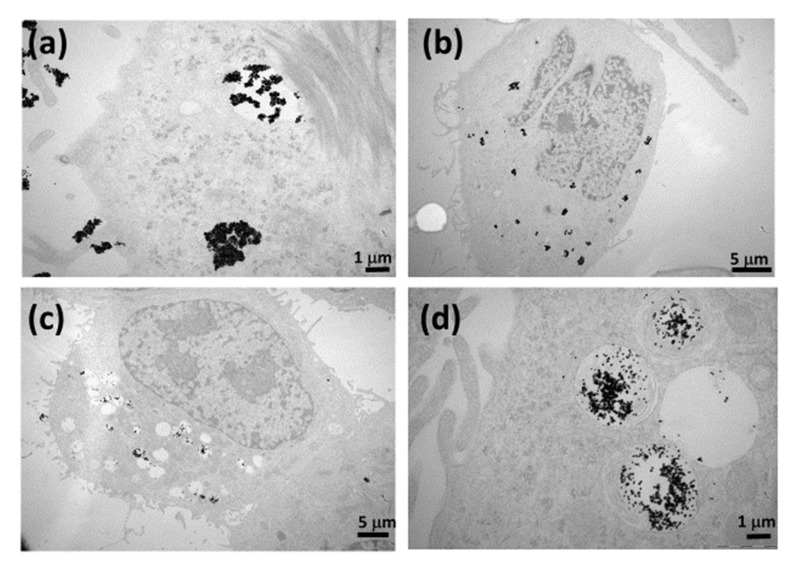
TEM images of the subcellular internalization of 21 µM concentration of curcumin–piperine gold nanogels in U-251 MG glioma cells; (**a**) curcumin–piperine NG interacting with the cell membrane and in a process to be up-taken by lysosome after 24 h of exposure; (**b**) curcumin–piperine NG is residing with the membrane-bound compartment after 48 h of exposure; (**c**) curcumin–piperine NG showing dissolved morphology and molecular dispersions found inside the cell cytoplasm after 72 h of exposure; (**d**) magnified image of curcumin–piperine NG residing with the membrane-bound compartment after 72 h of exposure.

Transmission electron microscopic image analysis was performed to visualize the subcellular localization of hybrid curcumin–piperine gold nanogel in U-251 MG cells after 48 h of exposure at 21 µM concentration under suspension culture conditions (37 °C, 5% CO_2_). The curcumin- and piperine-loaded gold nanogels are highly biocompatible and were internalized in glioma cells by endocytic pathways. The exposure of the glioblastoma cells to the gold nanogels results in the free movement of the hybrid drug-loaded gold nanogels outside of the cell’s surface. It was followed by the localization of the curcumin–piperine nanogel within a membrane-bound cell or cytoplasm (Figure 6a). At a later stage, interaction with the lysosome caused the nanogel to dissolve and release the gold nanoparticles (Figure 6b,c). The curcumin–piperine-loaded nanogel moved to reside with or assembled on the outer membrane of the membrane bound intracellular compartments (Figure 6d). This TEM image analysis elaborates the internalization efficiency of combinatorial drug-loaded gold nanogels to move from the lysosome to the inside of the vesicular compartments of U-251 MG cells to trigger drug release and cause cell death. The spherical gold nanoparticles do not cause cell apoptosis [22]. The cell death was caused by curcumin and piperine.

The gold nanogel is highly biocompatible, and its interaction with the cell membrane results in the triggering of endocytic pathways. It has been shown that piperine can be capsulated with different kinds of carriers for enhancement of dissolution as compared with pure piperine and the physical mixtures method [39]. The gold nanogel is not only biocompatible but also indicates an up-taking pathway and exhibits dispersal action in the lysosome and cell compartment.

## 4. Conclusions

Herein we report the synthesis of Lignin-g-p (NIPAM-co-DMAEMA) gold nanogels by using atom transfer radical polymerization. The nanogels were self-assembled into frozen micelles with a polystyrene core surrounded by a corona of PDMAEMA chains extending in the solvent. The curcumin and piperine were loaded into the gold nanogels to enhance their biodistribution and cytotoxic potential against the glioblastoma multiforme cancer cells. The prepared nanogels exhibited an average diameter of 180 nm with pH responsiveness. The kinetic drug release of hybrid curcumin–piperine gold nanogel was higher at 4 pH compared with the other combinations. The curcumin-loaded nanogel and piperine-loaded nanogel gave an average IC_50_ value of 30.72 μM and 35.04 μM, respectively. The curcumin–piperine hybrid nanogel gave the highest average cytotoxicity of all the drug-loaded nanogels with an average IC_50_ value of 21 μM. It was also confirmed that the combinatorial curcumin–piperine gold nanogels enter the glioma cells via lysosome endocytosis and disperse in the cytoplasm. Immunoblotting studies of U-251 MG cells elaborated that the F-actin protein induces the destabilization of the cytoskeleton of the glioma cancer cells which eventually triggers the caspase-3 apoptosis to cytotoxically kill glioma cancer cells. It was also confirmed that the combinatorial curcumin–piperine gold nanogels enter the glioma cells and reside inside the intracellular organelles to trigger cells’ death. The results of this study provided the experimental evidence to use combinatorial curcumin–piperine-loaded Lignin-g-p (NIPAM-co-DMAEMA) gold nanogels to inhibit the proliferation of glioblastoma multiforme cancer cells and to overcome the limitations of poor drug availability to cross the blood-brain barrier during the treatment of glioma cancer.

## Figures and Tables

**Table 1:** The average hydrodynamic diameter and zeta (ζ) potential values of various nano-formulations.

Nano-Formulations	Diameter (nm ± SD)	Zeta Potential (mV ± SD)
GNPs	15 ± 0.22	23.21 ± 0.18
Gold Nanogels	180 ± 12.35	23.50 ± 0.21
Curcumin-loaded gold nanogels	201 ± 12.26	26.43 ± 0.13
Piperine-loaded gold nanogels	198 ± 15.86	23.73 ± 0.60
Curcumin-Piperine-loaded gold nanogels	206 ± 12.56	29.67 ± 0.13

**Table 2:** Quantification of gold for cell-associated curcumin and piperine gold nanogels (21 µM) by using atomic absorption spectroscopy analysis on U-251 MG glioblastoma cells after day 3 of treatment (*n* = 3).

Nano-Formulations	Au Mass per Dish (×10^−1^ mg)	Cells per Dish ×10^6^	Estimate GNPs per Cell
Gold nanoparticles	1.41 ± 0.10	5.22 ± 0.15	8.02 ± 0.93 × 10^5^
Gold nanogel	1.46 ± 0.09	5.15 ± 0.09	8.61 ± 0.73 × 10^5^
Curcumin-loaded gold nanogels	1.14 ± 0.11	4.10 ± 0.20	8.49 ± 0.79 × 10^5^
Piperine-loaded gold nanogels	1.01 ± 0.07	3.70 ± 0.56	8.31 ± 0.89 × 10^5^
Curcumin–Piperine-loaded gold nanogels	0.69 ± 0.07	2.50 ± 1.09	8.41 ± 0.71 × 10^5^

Au density = 19.3 g/cc.

## Data Availability

Additional information is available in the supplementary file.

## Supplementary Materials

The following are available online at https://www.mdpi.com/article/10.3390/biomedicines9111516/s1, Figure S1: NMR spectrum example of PEG macroinitiator. PEG without end group modification, Figure S2: NMR spectrum example of PEG macroinitiator. PEG with end group modification, Figure S3: NMR spectrum of PDMAEMA block copolymer, Figure S4: NMR spectrum of PEG-PDMAEMA block copolymer, PEG-PDMAEMA, Figure S5: Images of curcumin and piperine loaded gold nanogels (a) curcumin-loaded gold nanogel was measured at a 488 nm laser wavelength (b) piperine-loaded gold nanogel was measured with a UV light source, Figure S6: Isobologram of curcumin-piperine hybrid nanogel combination.

## References

[1] Jain K.K. (2018). A Critical Overview of Targeted Therapies for Glioblastoma. Front. Oncol..

[2] Javed B., Ikram M., Farooq F., Sultana T., Mashwani Z.-U.-R., Raja N.I. (2021). Biogenesis of silver nanoparticles to treat cancer, diabetes, and microbial infections: A mechanistic overview. Appl. Microbiol. Biotechnol..

[3] Ikram M., Javed B., Raja N.I., Mashwani Z.-U.-R. (2021). Biomedical Potential of Plant-Based Selenium Nanoparticles: A Comprehensive Review on Therapeutic and Mechanistic Aspects. Int. J. Nanomed..

[4] Moorthi C., Kathiresan K. (2013). Curcumin–Piperine/Curcumin–Quercetin/Curcumin–Silibinin dual drug-loaded nanoparticulate combination therapy: A novel approach to target and treat multidrug-resistant cancers. J. Med. Hypotheses Ideas.

[5] Shim J.S., Lee J., Park H.-J., Park S.-J., Kwon H.J. (2004). A New Curcumin Derivative, HBC, Interferes with the Cell Cycle Progression of Colon Cancer Cells via Antagonization of the Ca^2+^/Calmodulin Function. Chem. Biol..

[6] Jäger R., Lowery R.P., Calvanese A.V., Joy J.M., Purpura M., Wilson J.M. (2014). Comparative absorption of curcumin formulations. Nutr. J..

[7] Bolat Z.B., Islek Z., Demir B.N., Yilmaz E.N., Sahin F., Ucisik M.H. (2020). Curcumin- and Piperine-Loaded Emulsomes as Combinational Treatment Approach Enhance the Anticancer Activity of Curcumin on HCT116 Colorectal Cancer Model. Front. Bioeng. Biotechnol..

[8] Choi B.H., Kim C.G., Bae Y.-S., Lim Y., Lee Y.H., Shin S.Y. (2008). p21Waf1/Cip1 Expression by Curcumin in U-87MG Human Glioma Cells: Role of Early Growth Response-1 Expression. Cancer Res..

[9] Bisht S., Feldmann G., Soni S., Ravi R., Karikar C., Maitra A., Maitra A. (2007). Polymeric nanoparticle-encapsulated curcumin (“nanocurcumin”): A novel strategy for human cancer therapy. J. Nanobiotechnol..

[10] Sabir F., Asad M.I., Qindeel M., Afzal I., Dar M.J., Shah K.U., Zeb A., Khan G.M., Ahmed N., Din F.-U. (2019). Polymeric Nanogels as Versatile Nanoplatforms for Biomedical Applications. J. Nanomater..

[11] Jain R.K., Stylianopoulos T. (2010). Delivering nanomedicine to solid tumors. Nat. Rev. Clin. Oncol..

[12] Javed B., Mashwani Z.-U.-R., Sarwer A., Raja N.I., Nadhman A. (2020). Synergistic response of physicochemical reaction parameters on biogenesis of silver nanoparticles and their action against colon cancer and leishmanial cells. Artif. Cells Nanomed. Biotechnol..

[13] Javed B., Mashwani Z.-U.-R. (2020). Synergistic Effects of Physicochemical Parameters on Bio-Fabrication of Mint Silver Nanoparticles: Structural Evaluation and Action Against HCT116 Colon Cancer Cells. Int. J. Nanomed..

[14] Ikram M., Javed B., Hassan S.W.U., Satti S.H., Sarwer A., Raja N.I., Mashwani Z.-U.-R. (2021). Therapeutic potential of biogenic titanium dioxide nanoparticles: A review on mechanistic approaches. Nanomedicine.

[15] Mangalathillam S., Rejinold N.S., Nair A., Lakshmanan V.K., Nair S.V., Jayakumar R. (2012). Curcumin loaded chitin nanogels for skin cancer treatment via the transdermal route. Nanoscale.

[16] Vashist A., Kaushik A., Vashist A., Bala J., Nikkhah-Moshaie R., Sagar V., Nair M. (2018). Nanogels as potential drug nanocarriers for CNS drug delivery. Drug Discov. Today.

[17] Aderibigbe B.A., Naki T. (2018). Design and Efficacy of Nanogels Formulations for Intranasal Administration. Molecules.

[18] Jo D.H., Kim J.H., Lee T.G., Kim J.H. (2015). Size, surface charge, and shape determine therapeutic effects of nanoparticles on brain and retinal diseases. Nanomed. Nanotechnol. Biol. Med..

[19] Langone P., Debata P.R., Inigo J.D.R., Dolai S., Mukherjee S., Halat P., Mastroianni K., Curcio G.M., Castellanos M.R., Raja K. (2014). Coupling to a glioblastoma-directed antibody potentiates antitumor activity of curcumin. Int. J. Cancer.

[20] Patial V., Mahesh S., Sharma S., Pratap K., Singh D., Padwad Y.S. (2015). Synergistic effect of curcumin and piperine in suppression of DENA-induced hepatocellular carcinoma in rats. Environ. Toxicol. Pharmacol..

[21] Bondaz L., Fontaine P., Muller F., Pantoustier N., Perrin P., Morfin I., Goldmann M., Cousin F. (2020). Controlled Synthesis of Gold Nanoparticles in Copolymers Nanomolds by X-ray Radiolysis. Langmuir.

[22] Tian F., Clift M.J., Casey A., del Pino P., Pelaz B., Conde J., Byrne H.J., Rothen-Rutishauser B., Estrada G., de la Fuente J.M. (2015). Investigating the role of shape on the biological impact of gold nanoparticles in vitro. Nanomedicine.

[23] He Z., Liu K., Manaloto E., Casey A., Cribaro G.P., Byrne H.J., Tian F., Barcia C., Conway G., Cullen P. (2018). Cold Atmospheric Plasma Induces ATP-Dependent Endocytosis of Nanoparticles and Synergistic U373MG Cancer Cell Death. Sci. Rep..

[24] Reeves A., Vinogradov S.V., Morrissey P., Chernin M., Ahmed M.M. (2015). Curcumin-encapsulating Nanogels as an Effective Anticancer Formulation for Intracellular Uptake. Mol. Cell. Pharmacol..

[25] Thorat B., Jangle R. (2013). Reversed-phase High-performance Liquid Chromatography Method for Analysis of Curcuminoids and Curcuminoid-loaded Liposome Formulation. Indian J. Pharm. Sci..

[26] Naksuriya O., Van Steenbergen M.J., Toraño J.S., Okonogi S., Hennink W.E. (2016). A Kinetic Degradation Study of Curcumin in Its Free Form and Loaded in Polymeric Micelles. AAPS J..

[27] Manaloto E., Gowen A.A., Lesniak A., He Z., Casey A., Cullen P.J., Curtin J.F. (2020). Cold atmospheric plasma induces silver nanoparticle uptake, oxidative dissolution and enhanced cytotoxicity in glioblastoma multiforme cells. Arch. Biochem. Biophys..

[28] Conway G., He Z., Hutanu A.L., Cribaro G.P., Manaloto E., Casey A., Traynor D., Milosavljevic V., Howe O., Barcia C. (2019). Cold Atmospheric Plasma induces accumulation of lysosomes and caspase-independent cell death in U373MG glioblastoma multiforme cells. Sci. Rep..

[29] Conway G., Zizyte D., Mondala J., He Z., Lynam L., Lecourt M., Barcia C., Howe O., Curtin J. (2021). Ursolic Acid Inhibits Collective Cell Migration and Promotes JNK-Dependent Lysosomal Associated Cell Death in Glioblastoma Multiforme Cells. Pharmaceuticals.

[30] Vinogradov S.V., Batrakova A.E.V., Kabanov A.V. (2004). Nanogels for Oligonucleotide Delivery to the Brain. Bioconjugate Chem..

[31] Muniz-Miranda M., Gellini C., Giorgetti E. (2010). Surface-Enhanced Raman Scattering from Copper Nanoparticles Obtained by Laser Ablation. J. Phys. Chem. C.

[32] Xie J., Pan X., Wang M., Yao L., Liang X., Ma J., Fei Y., Wang P.-N., Mi L. (2016). Targeting and Photodynamic Killing of Cancer Cell by Nitrogen-Doped Titanium Dioxide Coupled with Folic Acid. Nanomaterials.

[33] Dinari A., Abdollahi M., Sadeghizadeh M. (2021). Design and fabrication of dual responsive lignin-based nanogel via “grafting from” atom transfer radical polymerization for curcumin loading and release. Sci. Rep..

[34] Arvanitis C.D., Ferraro G.B., Jain R.K. (2020). The blood–brain barrier and blood–tumour barrier in brain tumours and metastases. Nat. Rev. Cancer.

[35] Thani N.A.A., Sallis B., Nuttall R., Schubert F.R., Ahsan M., Davies D., Purewal S., Cooper A., Rooprai H.K. (2012). Induction of apoptosis and reduction of MMP gene expression in the U373 cell line by polyphenolics in Aronia melanocarpa and by curcumin. Oncol. Rep..

[36] Sedeky A.S., Khalil I.A., Hefnawy A., El-Sherbiny I.M. (2018). Development of core-shell nanocarrier system for augmenting piperine cytotoxic activity against human brain cancer cell line. Eur. J. Pharm. Sci..

[37] Jeong S., Jung S., Park G.-S., Shin J., Oh J.-W. (2020). Piperine synergistically enhances the effect of temozolomide against temozolomide-resistant human glioma cell lines. Bioeng..

[38] Shoba G., Joy D., Joseph T., Majeed M., Rajendran R., Srinivas P.S.S.R. (1998). Influence of Piperine on the Pharmacokinetics of Curcumin in Animals and Human Volunteers. Planta Med..

[39] Thenmozhi K., Yoo Y.J. (2017). Enhanced solubility of piperine using hydrophilic carrier-based potent solid dispersion systems. Drug Dev. Ind. Pharm..

