# Supplementary material for: Enhanced Anticancer Response of Curcumin- and Piperine-Loaded Lignin-g-p (NIPAM-co-DMAEMA) Gold Nanogels against U-251 MG Glioblastoma Multiforme
Source: Biomedicines. 2021 Oct 21;9(11):1516. doi: 10.3390/biomedicines9111516 (PMC8615061; doi:10.3390/biomedicines9111516)
Supplement: Supplementary file 1 [file biomedicines-09-01516-s001.zip › biomedicines-1350991-supplementary.pdf]

biomedicines

MDPI

Enhanced Anticancer Response of Curcumin- and Piperin-Loaded Lignin-g-p (NIPAM-co-DMAEMA) Gold Nanogels Against U-251 MG Glioblastoma Multiforme

Supplementary materials

Figure S1

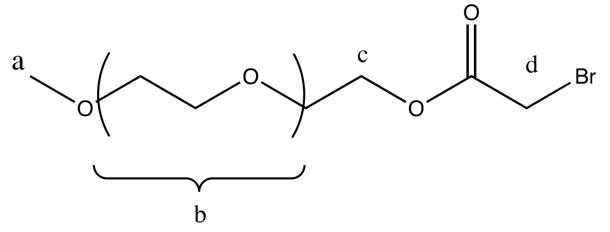

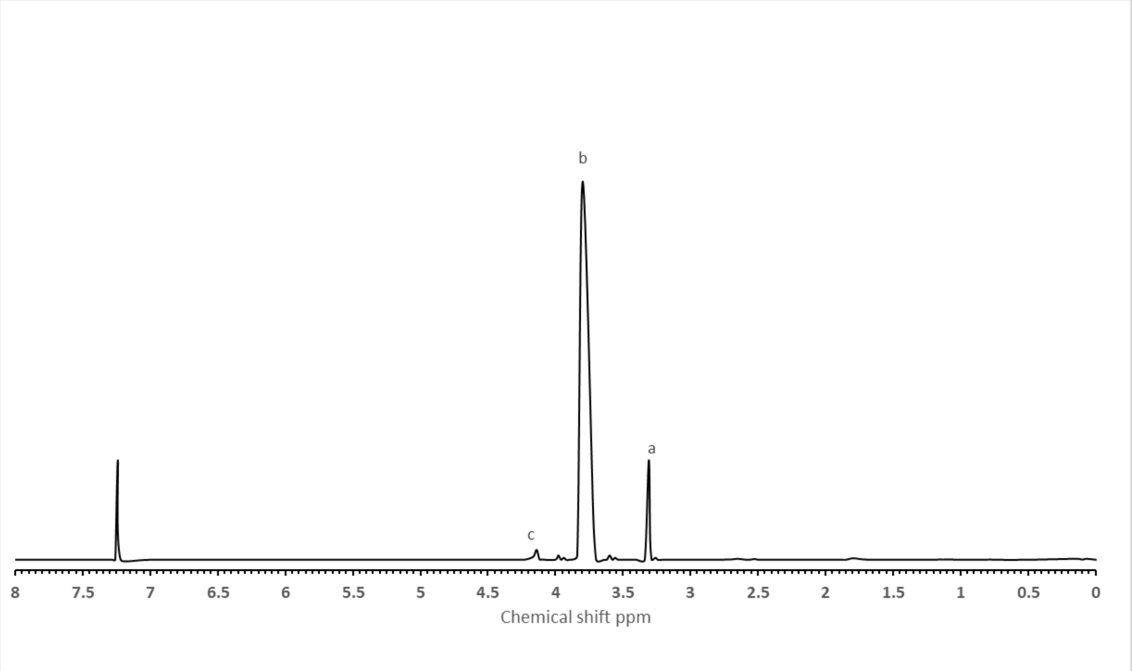

Figure S1. NMR spectrum example of PEG macroinitiator. PEG without end group modification.

Figure S2

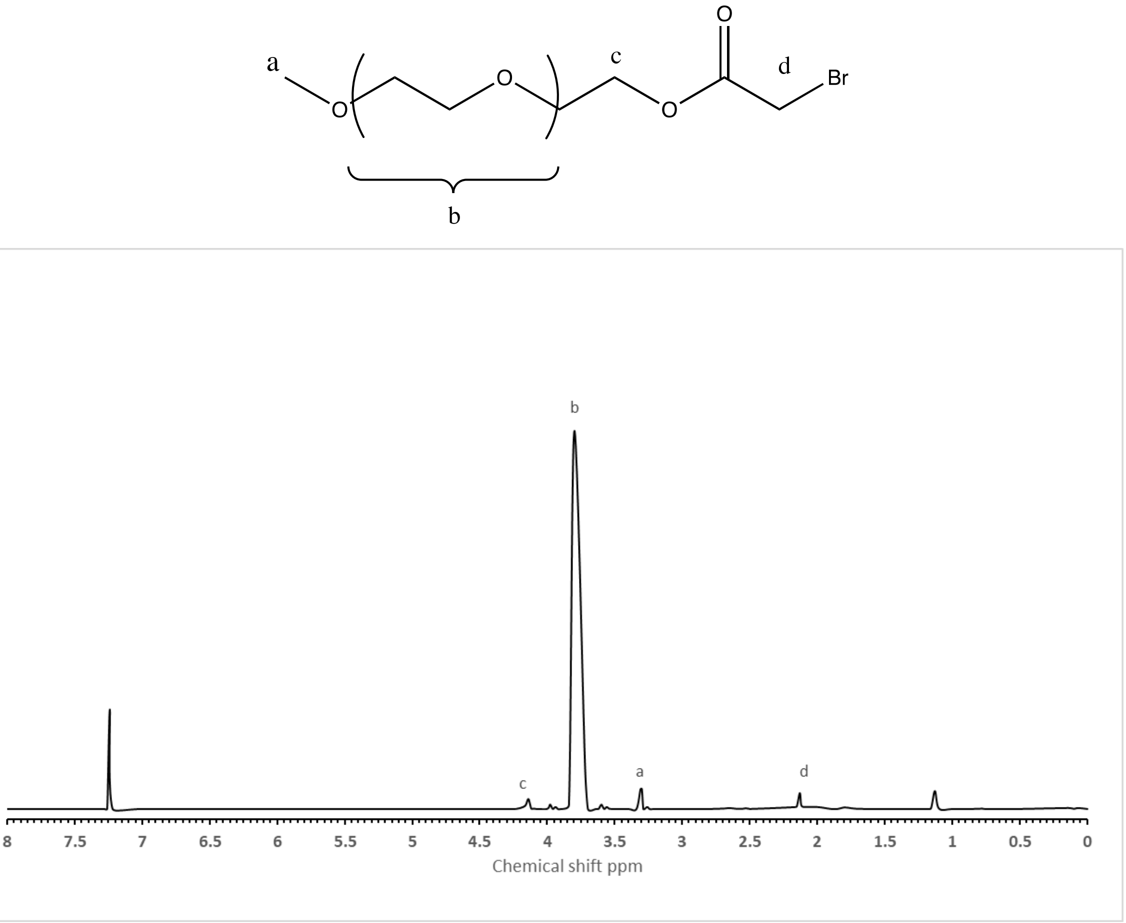

Figure S2. NMR spectrum example of PEG macroinitiator. PEG with end group modification.

Figure S3

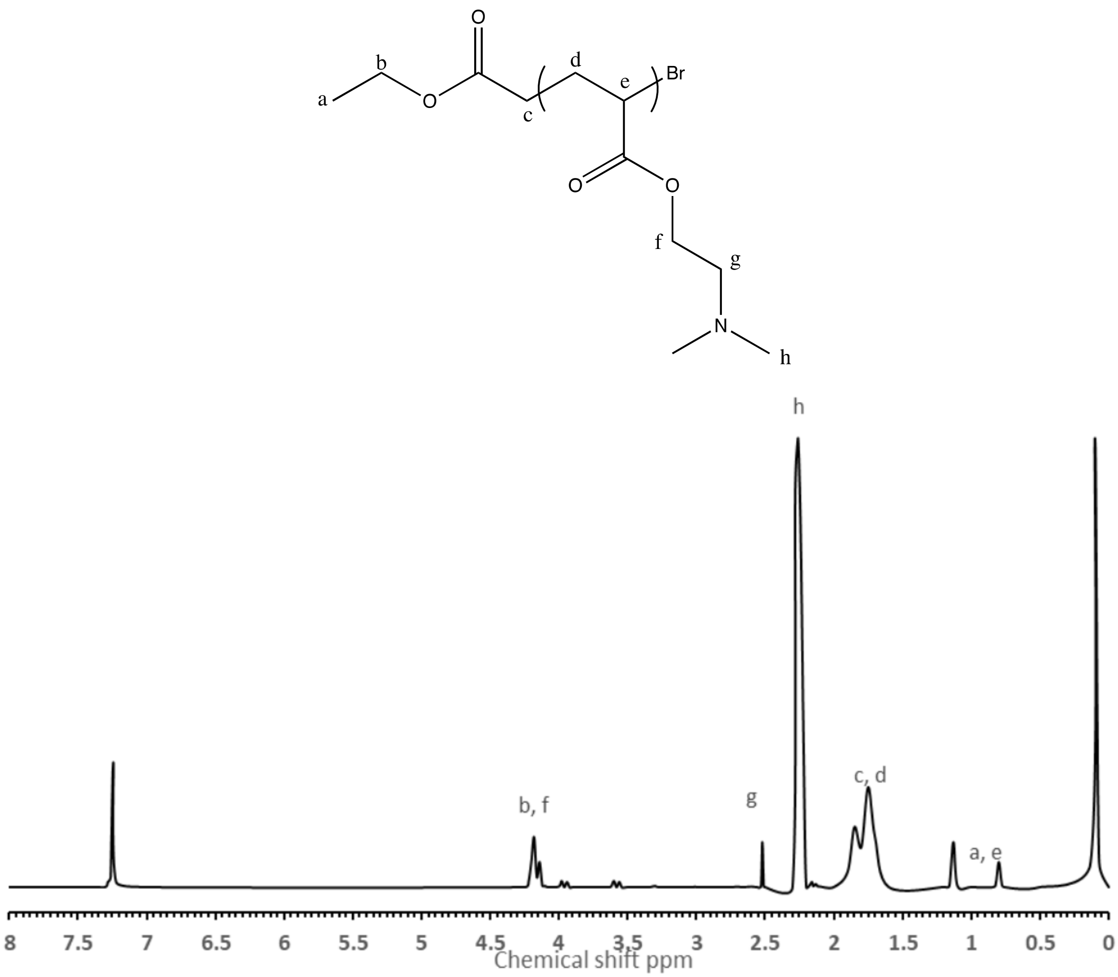

Figure S3. NMR spectrum of PDMAEMA block copolymer.

Figure S4

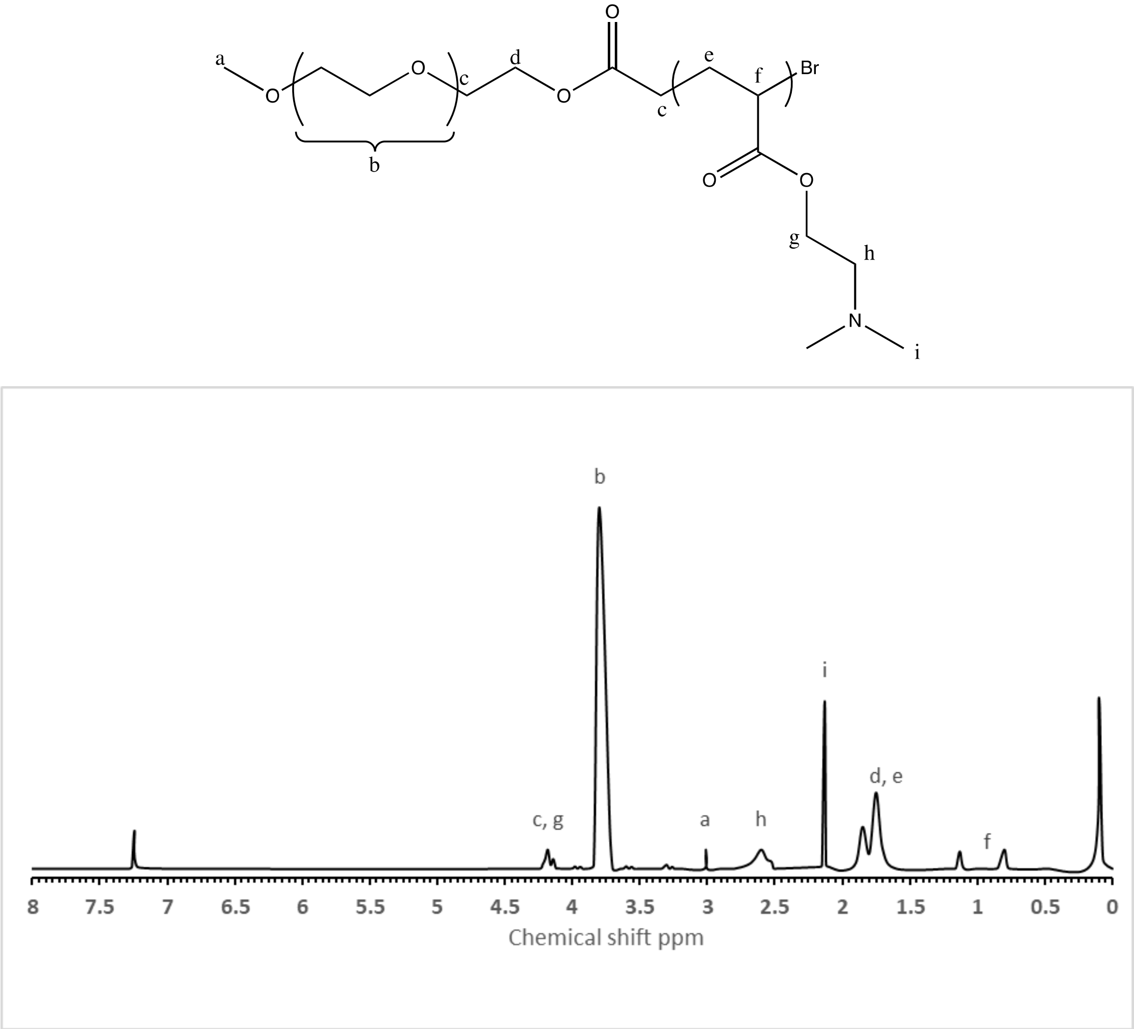

Figure S4. NMR spectrum of PEG-PDMAEMA block copolymer, PEG-PDMAEMA.

Figure S5

Gold nanogels were analyzed by using an Olympus epifluorescence microscopy with a 488 nm laser. A sample of 10 µL was added to a hemocytometer and a coverslip was loaded on the counting surface. Curcumin-loaded gold nanogel was measured at a 488 nm laser wavelength. Piperine-loaded gold nanogel was measured with a UV light source. Particle numbers were systematically counted in selected squares. The concentration of nanogel in the sample was multiplied by 10^4 particle concentrations per mL.

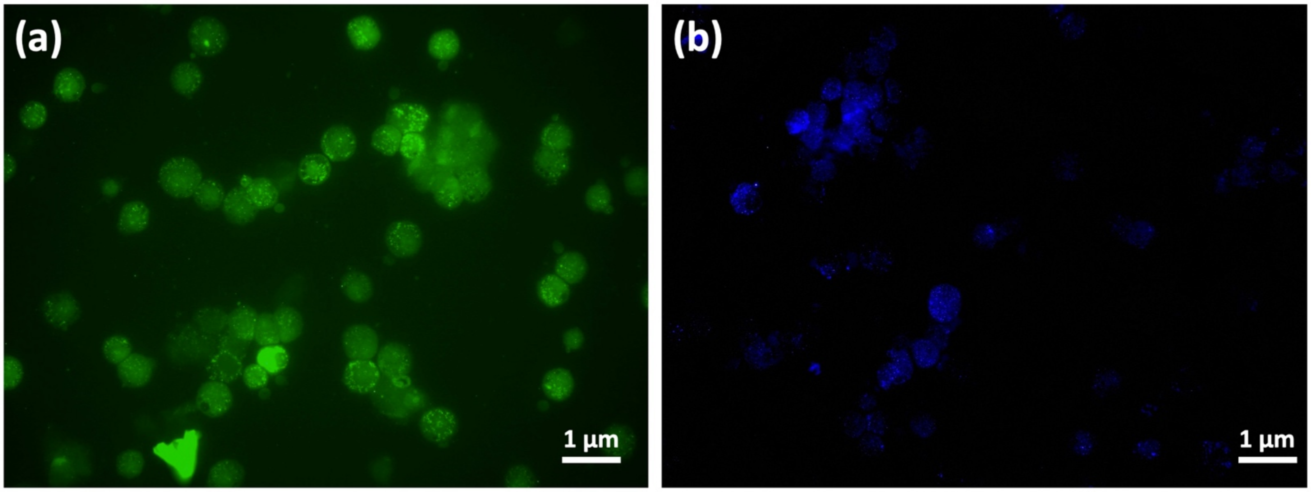

Figure S5. Images of curcumin and piperine loaded gold nanogels (a) curcumin-loaded gold nanogel was measured at a 488 nm laser wavelength (b) piperine-loaded gold nanogel was measured with a UV light source.

Figure S6

Isobologram analysis was performed to determine the antagonistic, synergistic or enhanced response of hybrid curcumin-piperine loaded nanogels against U-251 MG glioblastoma multiforme cancer cells. The combination index value was recorded at 0.886 which shows the mild synergistic and enhanced response of combinatorial curcumin-piperine loaded nanogels.

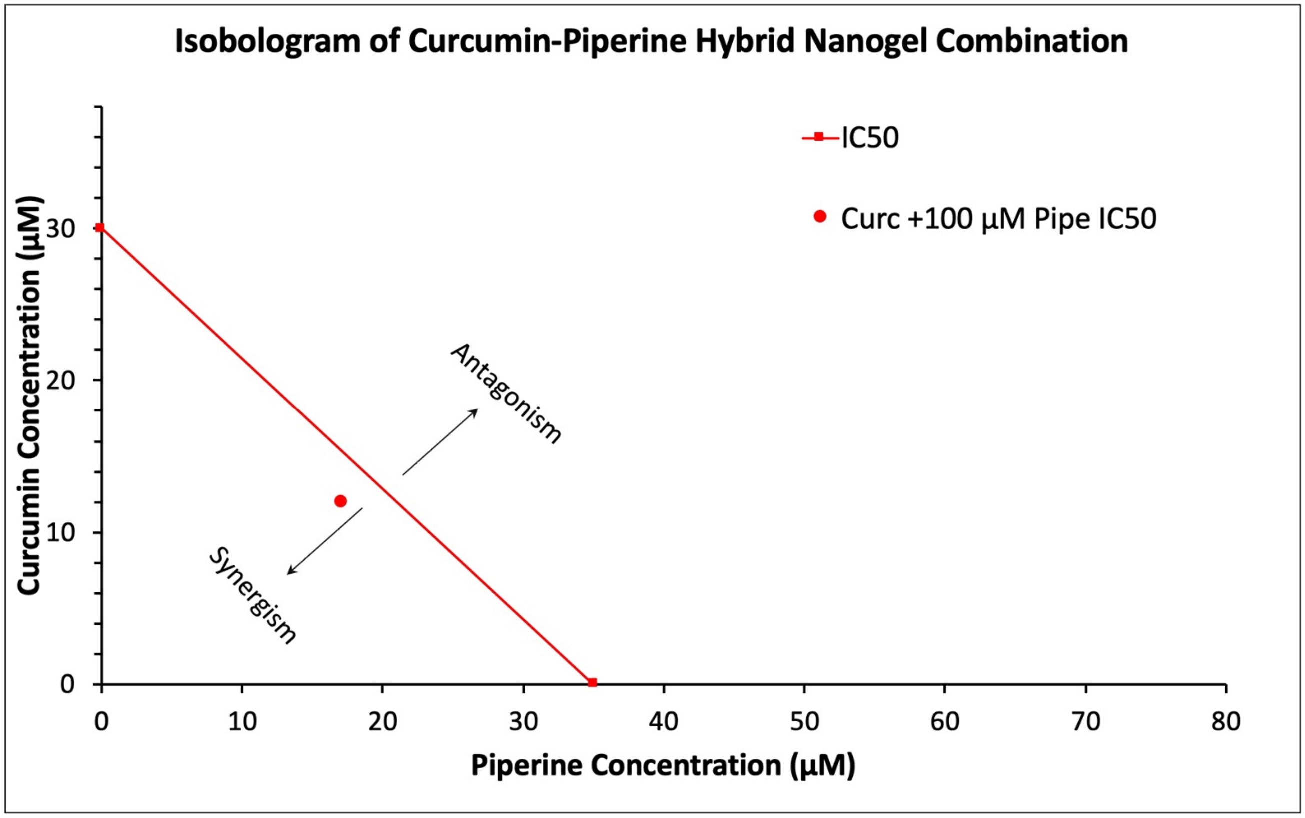

Figure S6. Isobologram of curcumin-piperine hybrid nanogel combination.
